# Place Cell-Like Activity in the Primary Sensorimotor and Premotor Cortex During Monkey Whole-Body Navigation

**DOI:** 10.1038/s41598-018-27472-4

**Published:** 2018-06-15

**Authors:** A. Yin, P. H. Tseng, S. Rajangam, M. A. Lebedev, M. A. L. Nicolelis

**Affiliations:** 1 Duke Center for Neuroengineering, Duke University, Durham, NC 27710 USA; 2 Department of Biomedical Engineering, Duke University, Durham, NC 27708 USA; 3 Department of Neurobiology, Duke University Medical Center, Durham, NC 27710 USA; 4 Department of Psychology and Neuroscience, Duke University, Durham, NC 27708 USA; 5 Edmond and Lily Safra International Institute of Neuroscience of Natal, Natal, 59066060 Brazil

## Abstract

Primary motor (M1), primary somatosensory (S1) and dorsal premotor (PMd) cortical areas of rhesus monkeys previously have been associated only with sensorimotor control of limb movements. Here we show that a significant number of neurons in these areas also represent body position and orientation in space. Two rhesus monkeys (K and M) used a wheelchair controlled by a brain-machine interface (BMI) to navigate in a room. During this whole-body navigation, the discharge rates of M1, S1, and PMd neurons correlated with the two-dimensional (2D) room position and the direction of the wheelchair and the monkey head. This place cell-like activity was observed in both monkeys, with 44.6% and 33.3% of neurons encoding room position in monkeys K and M, respectively, and the overlapping populations of 41.0% and 16.0% neurons encoding head direction. These observations suggest that primary sensorimotor and premotor cortical areas in primates are likely involved in allocentrically representing body position in space during whole-body navigation, which is an unexpected finding given the classical hierarchical model of cortical processing that attributes functional specialization for spatial processing to the hippocampal formation.

## Introduction

Little is known about the role of sensorimotor cortical areas, such as the primary motor (M1) and somatosensory (S1) cortices, in representing parameters of whole-body navigation. To date, neurophysiological mechanisms enabling whole-body navigation have been most extensively studied in the rat hippocampus^1–4^, where “place cells” representing spatial locations and navigation parameters were originally reported by O’Keefe and his colleagues^1,2^. Much less neurophysiological research has been conducted in nonhuman primates, employing virtual navigation tasks^5^ and whole-body motion^6–10^. In humans, neural correlates of navigation have been investigated using electroencephalography (EEG)^11,12^, neuroimaging^13,14^, and intracranial recordings^15–17^. Our recent studies in rhesus monkeys have shown that cortical motor (M1 and PMd) and somatosensory (S1) areas contribute to the encoding of body position during whole-body navigation^9,18^. This is surprising because, according to the classical hierarchical model of cortical processing^19–22^, space-coding should be restricted to the interactions between the hippocampus and association areas of the cortex.

To clarify the role of different brain areas in controlling whole-body navigation, we^9,18^ and others^8,23^ have recently started using a new experimental paradigm, where monkeys navigate in a room while seated in a motorized wheelchair. In these settings, monkeys can learn to control their wheelchair navigation^8,9,23^ using a brain-machine interface (BMI). In our implementation of the BMI for driving^9^, the velocity commands to the wheelchair were generated from the linearly combined activity of cortical neuronal ensembles recorded in the primary motor (M1), primary somatosensory (S1), and the dorsal premotor cortex (PMd). In the same study, we reported that a large fraction of cortical neurons in M1, S1, and PMd were tuned to the distance between the monkey and the target of navigation. This observation led us to hypothesize that monkey cortical neurons in these areas could also encode spatial location, which bears similarity to the encoding of space by the rodent place cell. Here, we report the results of testing this hypothesis. Our findings reveal that, in addition to representing arm reaching and wheelchair kinematics in egocentric coordinates, M1, S1 and PMd neurons allocentrically represent the monkey’s body location, as well as head and body orientation. Altogether, these findings suggest that hierarchically low cortical areas contribute to the representation of allocentric space during whole-body navigation.

## Results

**Figure 1:**
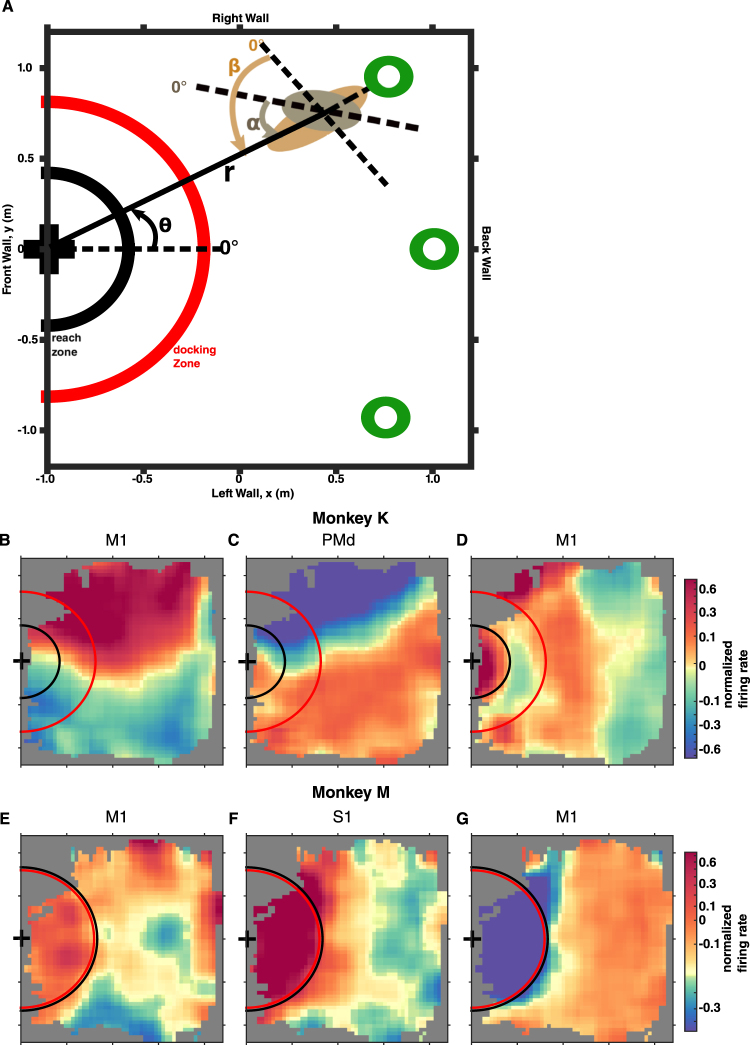
Wheelchair navigation setup and examples of spatially-selective neurons. (**A**) View from the top of the experimental room, where the monkey navigated in a motorized wheelchair under BMI control from one of three possible starting locations (shown as green circles) to the grape dispenser (marked by a ‘+’). The four walls of the experiment room are labeled as “front”, “back” “right” and “left”, which correspond to the monkey facing the grape dispenser and navigating from the back to the front of the room. Red semicircle labels the *docking zone* upon which an auto-pilot took over the wheelchair control. Black semicircle labels the *reach zone* within which monkeys initiated reach movements for the grape. The position of the monkey’s body and orientation of the head (gray ellipse) and trunk (tan ellipse) were evaluated using the following coordinates: $\theta$- the angle of the wheelchair’s location with respect to the dispenser; r – the distance from the wheelchair to the dispenser; $\alpha$– the angle of the dispenser location with respect to the monkey’s head direction; and $\beta$– the angle of the dispenser location with respect to the wheelchair’s direction. **(B**–**D)** Spatial tuning diagrams in representative neurons from monkey K and monkey M **(E**–**G)**, axis labels as in **(A)**. Color represents trial-average z-scored firing rates for different room locations. Cortical areas, where the neurons were recorded, are indicated.

Two monkeys (K and M) were employed in our experiments. During each recording session, a monkey sat in a motorized wheelchair and navigated from one out of three possible starting locations in a room towards the location of a grape dispenser (Fig. 1A). This whole-body navigation was performed under BMI control, where a linear decoding algorithm transformed cortical ensemble activity into translational and rotational velocity components responsible for the wheelchair movements^9^. The wheelchair passed through different room locations, which allowed us to construct position tuning maps that described how firing rates of individual M1, S1, and PMd neurons depended on wheelchair position (Fig. 1B–G). While most of the room was covered by the navigation trajectories, the coverage was not uniform for all locations, with the edges of the room being less visited. The distribution of the trajectories was also dependent on the wheelchair starting location – those starting at one side of the room tended to stay on that side. For analysis purposes, the region of the room where the grape dispenser was located was defined as the “front of the room” (Fig. 1A), whereas the “back of the room” corresponded to the region from which the wheelchair started to move. The “left” and “right parts of the room” corresponded to the monkey’s view when it faced the dispenser, the “front of the room”. We analyzed data from nine daily sessions in monkey K and 23 sessions in monkey M. Spike sorting was conducted on day one; afterwards the sorting parameters were adjusted when changes in neuronal waveforms were noticed. We analyzed activity patterns of 116 neurons in monkey K (27 in S1, 64 in M1, and 25 in PMd; see Supplementary Materials for selection criteria) and 124 neurons in monkey M (59 in S1 and 65 in M1).

### Individual neurons are tuned to both spatial location and orientation

Figure 1B–G shows that, in both monkeys, individual M1, S1, and PMd neurons modulated their firing rates according to the wheelchair spatial position. The position tuning of these neurons reflected not only the distance, *r*, from the wheelchair to the grape dispenser, but also whether the wheelchair was in the left or right part of the room. For example, the M1 neurons (from both monkeys) shown in Fig. 1B,E increased their firing rate when the wheelchair entered the right part of the room. Meanwhile, the PMd neuron shown in Fig. 1C (recorded in monkey K) showed an opposite pattern of position tuning: its firing rate decreased when the wheelchair entered the right part of the room and increased when it entered the left part. Figure 1D,F depicts an M1 neuron from monkey K and an S1 neuron from monkey M that, despite being very weakly tuned to the left-right dimension, were clearly tuned to the distance from the wheelchair to the grape dispenser: the firing rate increased in both neurons with decreasing distance. Figure 1G depicts an M1 neuron from monkey M whose firing rate decreased as the wheelchair approached the grape dispenser. In many neurons (see Fig. 1D–G), we observed a sharp change in firing rate that occurred when the wheelchair entered the “reach zone” (marked by black semi-circles in Fig. 1), from which the monkeys could reach for the food. Since monkey K was a smaller monkey, its reach zone was also smaller compared to the zone for monkey M.

In addition to the reach zone, Fig. 1 also shows the “docking zone” (marked by red circles). Inside this region, an autopilot program took over and maneuvered the wheelchair to dock it next to the dispenser to facilitate the monkey’s access to the grapes. For monkey M, the reach zone and the docking zone mostly coincided, since video analysis showed that this monkey began reaching while the wheelchair was being docked. Conversely, monkey K initiated reaching only when the wheelchair traveled close enough to the grape dispenser. This difference in the onset of reaching in the two monkeys was clearly visible in the neuronal patterns: a transition to reach-related activity occurred in monkey K only within the small reach zone (Fig. 1D), whereas in monkey M neuronal activity sharply changed when the wheelchair entered the docking zone (Fig. 1E–G). Overall, Fig. 1 shows that S1, M1 and PMd neurons in both monkeys were tuned to the 2D room position, including tuning in both left-right and back-front dimensions, while exhibiting, in addition, the well-known typical firing modulations related to arm reaching and hand grasping movements that have been classically associated with these cortical areas.

**Figure 2:**
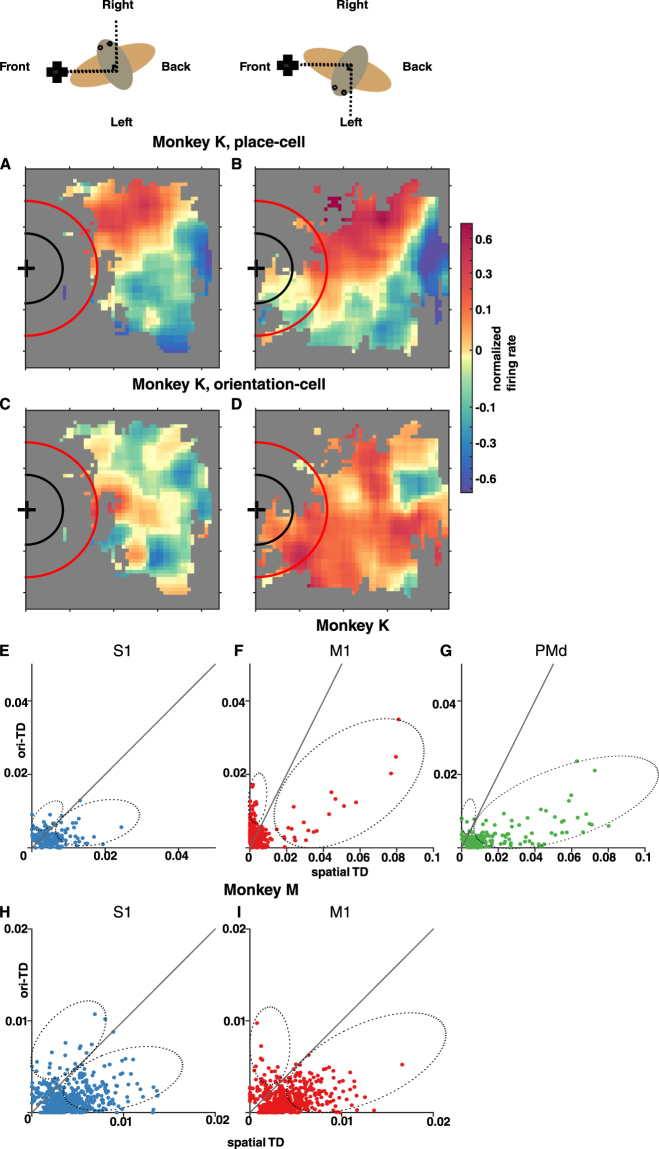
Neuronal tuning to room location and head orientation. (**A**–**D**) Modulation of spatial tuning patterns by head orientation in two neurons from monkey K. Conventions as in Fig. 1B–G. The color plots correspond (see key on top) to the right from the grape dispenser (*α* from 45 to 135 degrees; **A**, **C**) or to the left from the dispenser (α from −45 to −135 degrees; **B**, **D**). The spatial tuning pattern of one PMd neuron did not substantially change with the head orientation (**A**,**B**), whereas the pattern of another M1 neuron dramatically changed (**C**,**D**). **(E**–**I)** Scatterplots of tuning depth (TD) to room location versus TD to orientation (α and *β*) for monkey K’s (**E–G**) or monkey M’s (H-I) neurons with significant mutual information to either parameter from all sessions, across cortical areas: S1 (**E**, **H**), M1 (**F**, **I**) and PMd (**G**). The diagonal lines show where space-TD equals to orientation-TD. The dashed ellipses below and above the diagonal lines illustrate clusters of highly-tuned position-preferring and orientation-preferring neurons, respectively.

Further analysis revealed that, in addition to representing room position, the firing rates of M1, S1, and PMd neurons were affected in varying degrees by the direction relative to room landmarks in which the monkeys turned their heads and the rotations of the wheelchair. We named this type of neuronal tuning “orientation-related”. To illustrate orientation tuning, Fig. 2A–D compares the instances when the monkey’s head pointed to the right of the grape dispenser (45 < *α* < 135, Fig. 2A,C) to the instances when the monkey’s head pointed to the left of the dispenser (−135 < *α* < −45, Fig. 2B,D). Two neurons from monkey K are illustrated: a PMd neuron exhibited a strong preference to the right part of the room, irrespective of where the monkey’s head pointed (Fig. 2A,B), while an M1 neuron displayed firing patterns that were clearly different in these two cases; when the monkey’s head pointed to the right of the dispenser (Fig. 2C) this neuron’s firing rate was much lower than when the monkey’s head pointed to the left (Fig. 2D). In general, we observed that M1, S1, and PMd neurons represented both room location and monkey head orientation, with some neurons being more location-tuned and others more orientation-tuned.

To estimate the proportion of neurons that show significant tuning to spatial location and body/head orientation, we conducted a mutual-information analysis^10,24,25^ that evaluated the correspondence between neuronal rates and individual task covariates (spatial location, monkey head angle *α*, and wheelchair angle *β*; Fig. S1 and Table S1). In monkey K, significant mutual information (permutation test with FDR correction, p < 0.05) to at least one of these three covariates was found for 80.4 ± 12.9% neurons (mean ± std across sessions) (73.3 ± 21.1% in S1, 84.7 ± 11.6% in M1, and 76.9 ± 12.3% in PMd). In monkey M, 47.5 ± 18.7% neurons were tuned to at least one parameter (47.2 ± 18.4% in S1 and 47.9 ± 19.5% in M1). Tuning to room position was found for 64.0 ± 18.8% (63.0 ± 20.6% in S1, 62.8 ± 20.3% in M1, and 68.0 ± 15.7% in PMd) and 36.5 ± 16.1% (35.4 ± 14.7% in S1, and 37.4 ± 17.7% in M1) in monkey K and monkey M respectively.

To quantify the dependence of the firing rate of individual neurons on position and rotational covariates, we pulled out the neurons with significant mutual information to position or orientation parameters. Next, we applied generalized additive models^26,27^ (GAM) to these neurons. This analysis was complicated by the fact that orientation and position parameters were mildly correlated (Pearson correlation coefficient <0.3 for all sessions). To cope with this issue, we included all second-order interaction effects between covariates. We defined the neuronal tuning depth (TD) of a neuron to a covariate or a group of covariates as the fraction of that neuron’s firing rate deviance uniquely explainable by that covariate or group of covariates (see Methods).

To assess the composition and properties of the neuronal populations tuned to room position and wheelchair and head orientation, we grouped the covariates of our GAM into position-related (x and y) and orientation-related (α and β). Next, for each neuron entered into the GAM analysis, we calculated its position and orientation neuronal TD, respectively, and plotted position TD against the orientation TD values (Fig. 2E–I). In these scatter-plots, each point represents an assessed neuron with significant TD (F-test, p < 0.005) to at least one parameter. The scatter-plots showed that many M1, S1, and PMd, neurons were tuned to both room position and orientation parameters. Of the neurons with significant mutual information to position or orientation parameters that were used in the GAM analysis, 30.4 ± 17.6% (mean ± std across sessions) in monkey K (S1, M1, and PMd) and 25.7 ± 18.5% in monkey M (S1 and M1) showed significant tuning to both parameters (see Table S2–3). While representing both position and orientation, neurons could still be classified as position-preferring versus orientation-preferring (above and below the diagonal lines, respectively, in Fig. 2E–I). Additionally, for each monkey, we defined highly tuned neurons as those with preferred TD (for position or orientation) exceeding the median TD for the neuronal sample that entered the GAM analysis in the corresponding recording sessions. The proportions of highly tuned neurons and neurons with preferences for either position or orientation tuning varied according to cortical area (S1, M1, or PMd) and monkey (K or M). In both monkeys, the distribution of TDs was positively skewed because of the presence of highly-tuned neurons (Supplementary Fig. S3). In monkey K, the TDs for highly tuned neurons, preferring either location or orientation, were markedly stronger (Kruskal-Wallis, p < 0.001; α = 0.05 for Tukey’s multiple comparison; on TDs pooled over all sessions) for PMd neurons (0.011 ± 0.020; session pooled median ± interquartile range) than for M1 neurons (0.005 ± 0.003) and S1 neurons (0.006 ± 0.004). The GAM showed that most neurons were position preferring in PMd, where they constituted 81.9 ± 6.5% (mean ± std) of all neurons and 91.5 ± 5.0% of highly tuned neurons. In M1, 32.1 ± 19.0% of all neurons and 44.8 ± 13.1% of highly tuned neurons were position-preferring. In S1, 53.1 ± 16.8% of all neurons and 71.9 ± 16.6% of highly tuned neurons were position-preferring. The percentage of all neurons that were position-preferring in PMd was significantly higher than in M1 (Friedman’s test, p < 0.001; α = 0.05 for Tukey’s multiple comparison) but not than in S1. The percentages of highly-tuned neurons that were position-preferring in both PMd and S1 were significantly higher than in M1 (Friedman’s test, p < 0.001; α = 0.05 for Tukey’s multiple comparison). Although M1 had fewer position-preferring neurons, a clear cluster of neurons with high TD for position was found in this area (marked by an ellipse in Fig. 2F). In monkey M, preferred TDs were lower compared to monkey K (p < 0.005, Wilcoxon rank-sum test, neurons pooled over sessions; see also Fig. S3), and position-preferring and orientation-preferring neurons were approximately equally represented in both M1 and S1 (Friedman’s test; See also Table S3).

### Population tuning characteristics

**Figure 3:**
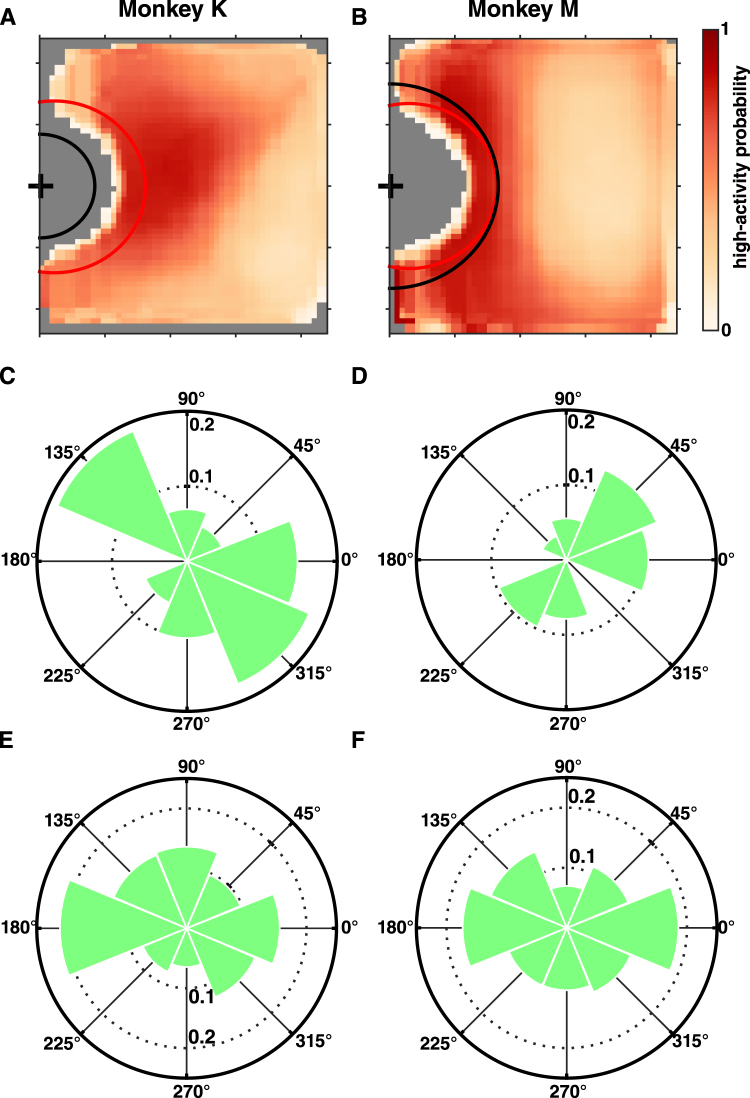
Place fields and preferred-directions for the neuronal ensembles. (**A**,**B**) Heatmaps of ensemble-average place fields for monkey K (**A**) and monkey M (**B**); plots represent averages across all sessions. Only position-preferring neurons were included. Each neuron’s place field was defined as the room locations where the neuron’s tuning function (as fitted by GAM) exceeded the median value over all room locations. A histogram of place-fields was constructed from all position-preferring cells’ place-fields and normalized by the number of position-preferring cells in each session. These session histograms were then averaged to produce the final heatmap. (**C**,**D)** Circular histograms of preferred $\alpha$ directions averaged over sessions for monkey K (**C**) and monkey M (**D**). For each session, the preferred $\alpha$ directions of all orientation-preferring neurons were extracted from their $\alpha$-tuning curves (as fitted by GAM). The preferred directions were then represented as circular histograms. Radial direction represents proportion of neurons. **(E**,**F)** Circular histograms for $\beta$. Conventions as in (**C**,**D**).

To visualize the position-tuning properties for the populations of position-preferring neurons, we plotted the session average heatmaps that represented ensemble-average place fields for the position-preferring neurons; only the room locations outside the docking zone are shown (Fig. 3A,B). For each neuron, a place field was defined as the room locations where the neuron’s tuning function fitted by GAM exceeded the median value over all room locations. For both monkeys, the heatmaps revealed non-uniform distributions of neuronal place fields (Kolmogorov-Smirnov test, p < 0.01), with gradients of neuronal activity in back-front and left-right dimensions. In monkey K neuronal place fields were concentrated in the front-right part of the room. To visualize orientation tuning, we plotted the average histogram of the preferred α (monkey head angle) and β (wheelchair angle) directions for the orientation-preferring cells over all sessions (Fig. 3C–F). A diversity of preferred directions was found (Kolmogorov-Smirnov test, p < 0.01) but their distributions were different, with peaks around 135 degrees (i.e. facing away from the grape dispenser) for both α and β in monkey K (Fig. 3C,E), and peaks around 0 and 180 degrees (i.e. facing either toward or away from the grape dispenser) for α and β in monkey M, respectively (Fig. 3D,F).

### Spatial tuning properties across different days

**Figure 4:**
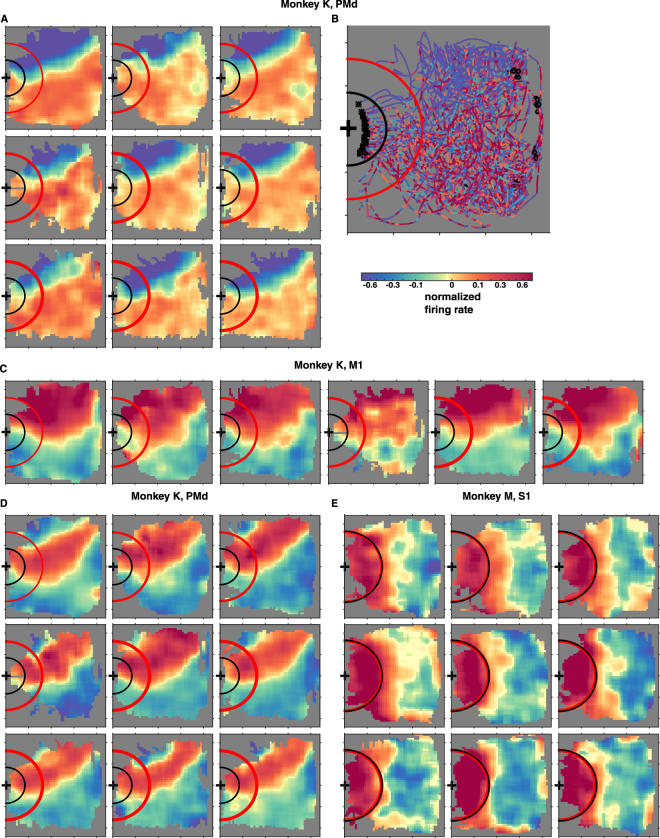
Individual neurons with consistent position tuning across multiple sessions. (**A**) The position-tuning diagram of monkey K’s PMd neuron shown in Fig. 1C for all nine experimental sessions. Session ordered from left to right, then top to bottom. The first session displayed corresponds to Fig. 1C. (**B**) Sample brain-controlled navigation trajectories for the same session as the data shown in Fig. 1C. Colors represent normalized firing rates along the trajectories. Black ‘*‘ mark the location at the end of each trial, and black ‘o’ mark the starting locations of each trial. **(C)** The position-tuning diagram of monkey K’s M1 neuron shown in Fig. 1B, for the first six experimental sessions. Session number increases from left to right. The first session is the same as shown in Fig. 1B. (**D**) Position tuning diagrams of monkey K’s PMd neuron for all nine experimental sessions. Session ordered from left to right, then top to bottom. **(E)** Position tuning diagrams of monkey M’s S1 neuron shown in Fig. 1F for nine consecutive experimental sessions, just before the session shown in Fig. 1F. Session ordered from left to right, then top to bottom.

By recording from the same sample of neurons over several daily sessions, we observed that the positional and orientation tuning properties of individual cells often remained the same over multiple days. Figure 4A illustrates that the position tuning of monkey K’s PMd neuron, the same as the one shown in Fig. 1C, remained stable over nine recording sessions. This neuron consistently increased its firing rates when the wheelchair entered the left side of the room (also see Fig. 4B for modulations in the firing rate of the same neuron along individual navigation trajectories). Figure 4C illustrates the position tuning of monkey K’s M1 neuron shown in Fig. 1B, which remained stable over six recording sessions. This neuron increased its firing rates when the wheelchair was on the right side of the room. Figure 4D illustrates the nine-day stability of neuronal spatial patterns in a highly-tuned position-preferring PMd neuron from monkey K, which increased its discharge rates as the wheelchair entered the right side of the room but then decreased firing close to the right wall. Figure 4E illustrates the nine-session stability of neuronal place-fields patterns in a S1 neuron from monkey M, which increased its discharge rates as the wheelchair entered the front of the room.

To estimate the proportion of cells with stable spatial tuning, we first selected neurons from each monkey that were highly tuned for at least 70% of all recording sessions. This selection assured that we analyzed only very stable neurons (in terms of the presence of position tuning) and disregarded the less stable units. In the subsequent analyses, all of the selected neurons were considered even if some of them did not have a significant MI in a given session. About 35.3% in monkey K (33.3% in S1, 34.4% in M1, and 40.0% in PMd) and 12.1% in monkey M (15.3% in S1 and 9.2% in M1) fulfilled this criterion. Using this subset of cells, we then quantified the similarity between the neurons’ tuning patterns from pairs of sessions as their correlation coefficients (i.e. between-session consistency). Correlation coefficient values were computed similarly between halves of the same session (i.e. within-session consistency, see Methods). All correlation values were significantly different from zero (permutation test, p < 0.001) and there was no significant difference between within- and between-session correlation values (Wilcoxon rank-sum test). Thus, the across-session variability of neuronal patterns was the same as the within-session variability, indicating that neuronal spatial tuning properties stayed unchanged across multiple days.

### Decoding of position

**Figure 5:**
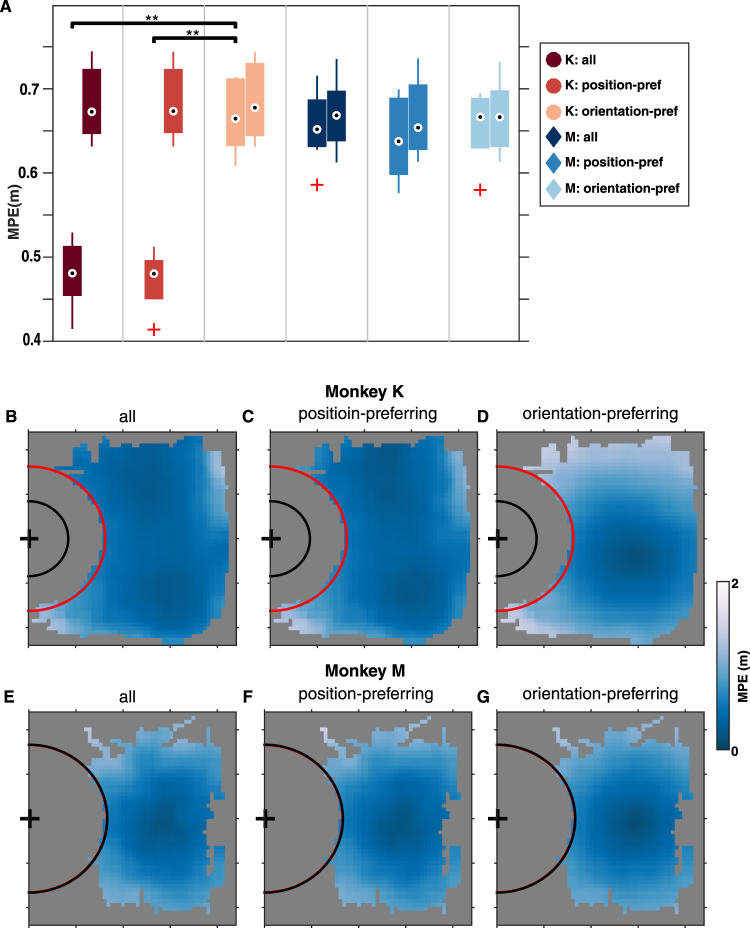
Decoding of room location from neuronal ensemble activity. (**A**) Box plots showing the distribution of decoding accuracy and chance decoding accuracy (left and right boxes within each group, respectively) measured by the mean prediction error (MPE) in meters, in monkey K and monkey M using different population of neurons. Only sessions with decoding accuracy significantly above chance are shown. The distributions of chance-level decoding accuracy are constructed from the lower-end of the 95% confidence interval of the selected sessions’ chance-level error. (**) indicates significant p-value < 0.01 for post-hoc Tukey’s multiple comparison following Kruskal-Wallis test. **(B–G)** Color plots of MPE as a function of room location in one representative session each for monkey K (**B–D**) and monkey M (**E–G**) – from using all neurons (**B**,**E**), position-preferring neurons (**C**,**F**), and orientation-preferring neurons (**D**,**G**).

The position tuning properties of the recorded neurons allowed us to conduct offline decoding of the wheelchair’s position using neural network decoders. All these decoders had one hidden-layer and used spiking activities from either all neurons with significant MI (*all*), position-preferring neurons (*position-pref*), or orientation-preferring neurons (*orientation-pref*). The decoder performance was evaluated by calculating the mean prediction error (MPE) in meters, defined as the average distance between the decoded and actual locations pooled over the experiment room (Fig. 5A). In monkey K, *all* and *position-pref* decoders performed significantly better than chance for all sessions, *orientation-pref* decoders performed better than chance for 7 out of 9 sessions (permutation test, p < 0.05). The *all* and *position-pref* decoders significantly outperformed *orientation-pref* decoders (Kruskal-Wallis, p < 0.01; α = 0.05 for Tukey’s multiple comparison). Figure 5B–D shows MPE at different room positions for a representative session of monkey K. The *all* and *position-pref* decoders predicted location with low errors over large areas of the experimental space (Fig. 5B,C). The *orientation-pref* decoders performed well near the center of the room, but poorly around the left and right boundaries (Fig. 5D). In monkey M, *all* and *orientation-pref* decoders performed significantly better than chance for 11 out of 23 sessions, *position-pref* decoders performed significantly better than chance for 10 out of 23 sessions (permutation test, p < 0.05). Notably, the average trial durations in sessions with significant decoder performance were significantly shorter than those in sessions with chance-level decoder performance (p < 0.01, Wilcoxon rank-sum). All three types of decoders had similar performances, with the *position-pref* decoders yielding marginally better predictions (Fig. 5A). Monkey M’s decoders all predicted position well near the room center but poorly near room boundaries (Fig. 5E–G). Better decoding performance in monkey K compared to monkey M is not surprising, since monkey K’s neurons generally had higher TD to position than monkey M. Thus, a higher amount of position information translated into higher decoding performance.

Orientation parameters (α and β) were decoded similarly as well using *all*, *position-pref*, and *orientation-pref* decoders. The decoding performances measured in Pearson’s product-moment correlation coefficient (cc) are shown in Figure S4. In monkey K, prediction of both orientation parameters was significantly better than chance for all of the sessions for all types of decoders used. The prediction of α by *all* and *orientation-pref* decoders were significantly better than that by *position-pref* decoders (Kruska-Wallis, p < 0.05 and p < 0.01, respectively; α = 0.05 for Tukey’s multiple comparison). In monkey M, prediction of α was significantly better than chance for all sessions with *all* and *position-pref* decoders, and for 20 out of 23 sessions with *orientation-pref* decoders. Prediction of α by *all* decoders were significantly better than that by *orientation-pref* decoders (Kruskal-Wallis, p < 0.01; α = 0.05 for Tukey’s multiple comparison). Prediction of β was significantly better than chance for all sessions with *all* and *position-pref* decoders, and for 22 out of 23 sessions with *orientation-pref* decoders. Prediction of β by *all* decoders were significantly better than that by *orientation-pref* decoders (Kruskal-Wallis, p < 0.01; α = 0.001 for Tukey’s multiple comparison).

### Decoding of arm reach state

As the wheelchair entered the reach zone, monkeys initiated reach movements for the grape. Population perievent time histograms aligned on the wheelchair entering the reach zone clearly showed reaching-related neuronal activities (Fig. S5). We classified the presence of reaching movements from these patterns of neural activities using decision-tree ensemble classifiers. Figure S5 shows the classification results in both monkeys as heatmaps of reach-state probability in different parts of the room for representative sessions. Classification was performed using different populations of neurons: all neurons with significant MI (Fig. S5C,F), subpopulations of position-preferring neurons (Fig. S5D,G) or subpopulations of orientation-preferring neurons (Fig. S5E,H). The classifier correctly recognized the reaching state in the smaller reach zone for monkey K (Fig. S5C–E) and the larger zone for monkey M (Fig. S5F–H). As the number of units with significant MI, position- and orientation-preferring units varied across sessions, we plotted in Supplementary Fig. S5I the accuracy of classifiers as a function of the ensemble size. The accuracy was quantified by Cohen’s *κ*^28^, a metric that is appropriate for measuring classification accuracy on unbalanced datasets. All of monkey K’s classifiers and approximately half of monkey M’s classifiers, using all units with significant MI, achieved good accuracy (0.61 < *κ* < 0.80, marked by the gray band) on the Landis and Koch scale^28^. In both monkeys, the classifiers’ performance increased with the ensemble size. This dependence was significantly different for the neuronal populations in monkey M (nonparametric ANCOVA, p < 0.001), but not in monkey K (nonparametric ANCOVA)^29^. For both monkeys, after taking into account the sample size, the classification accuracy did not significantly differ by subpopulation type (nonparametric ANCOVA).

## Discussion

In the present study, two rhesus monkeys employed a BMI to control the movements of a motorized wheelchair in order to navigate to a fixed target location in a room. This experimental setting allowed us to study cortical representation of the animals’ body position and orientation. We observed that a significant fraction of S1, M1, and PMd neurons exhibited spatial tuning, with both position and orientation encoded by neuronal discharges. These neuronal patterns resembled two classes of neurons previously reported in the hippocampus: place cells^1–4^ and head-direction cells^30,31^. Additionally, the spatial tuning properties of cortical neurons remained stable over several days, which is also similar to the place-fields observed in the hippocampal place-cells^2,32^. Since we did not record from the hippocampus, it remains to be examined to what extent space-representing neuronal activity that we observed in navigating monkeys would match the hippocampal activity during the same task. We leave this question for further studies.

There are several potential explanations that could account for our findings, different from cortical encoding of body position and orientation *per se*. For example, neurons in the sensorimotor and premotor cortical areas have been shown to encode rewards^33,34^, so it is possible that expectation of reward could explain neuronal tuning to the distance from the monkey to the grape dispenser^9^ and the front-back position-selectivity. Yet, position selectivity in the left-right dimensions is inconsistent with this hypothesis. Reward expectation depends on the distance from the monkey to the reward, but not on the left-right dimension. Even if reward expectation affected the neuronal rates in our experiments, a similar result has been reported for monkey hippocampus^35^. In this latter study, the authors concluded that “the primate hippocampus contains a representation of the reward associations of places”. Our findings may be also interpreted in a similar way: activity of M1, S1, and PMd neurons represented the 2D location of the monkey’s body with respect to reward.

Motor preparatory activity^36^ is another possible alternative explanation for our findings. One could suppose that monkeys were preparing arm reaching movements in different directions, while traveling in the wheelchair, for example leftward reaching when the wheelchair was close to the right wall of the room and rightward reaching when the wheelchair was near the left wall. The flaw with this potential explanation is that our monkeys never performed these “eventual” arm reaching movements in different directions. Instead, since these animals were over trained, in every session we noticed that only after the wheelchair was docked and the monkeys faced the grape dispenser did they produce a comfortable and stereotyped arm reach. Invariably, these arm reaches occurred only in the forward direction, relative to the monkeys’ body. As such, reach-related activity could not account for the tuning to position. In monkey K, position tuning in the left-right direction was especially strong. Yet, this monkey always initiated an arm reach after the wheelchair was securely docked and the movement was always in the forward direction. Conversely, monkey M often initiated arm reaching movements before the wheelchair was docked, but its neurons were poorly tuned in the left-right dimension during the navigation. Based on these considerations, we conclude that motor activity related to arm reaching movements offers a very unlikely explanation for the neuronal position encoding observed here.

A more exotic explanation would involve a “virtual arm” that extends from the monkey to the food while the monkey navigates. This explanation would resemble Rizzolatti’s premotor theory of attention^37^ where a movement in a certain direction is prepared but never executed. This hypothesis, however, does not explain why a neuron remains tuned to the same spatial location when the chair is rotated.

Another hypothesis is that the position-tuning patterns observed were related to planning and executing a whole-body trajectory in space. This explanation does not contradict the characterization of our findings in terms of place cells; rather, it could provide an explanation for the function of place-cell tuning in M1, PM, and S1. The explanation is also consistent with previous theories, since it has been suggested that allocentric spatial information from the hippocampus-entorhinal circuits is projected to the parietal cortex, where it is transformed into body-centered coordinates and then relayed to the motor and premotor cortices for execution^22^. Yet, if this were true, a prevalence of egocentric encoding would be expected for M1, S1 and PMd, which we did not observe in our experiments.

In the future, it will be interesting to test whether the cortical place fields reported here rotate, like observed for hippocampal place-fields, following rotations of environmental cues^38,39^. While this possibility has yet to be thoroughly tested, we have recently reported that place-fields of M1 and PMd neurons also change when the objects in the room are moved^18^. In that study, one monkey (the Passenger) was carried by a wheelchair while a second monkey (the Observer) sat in a corner of the room observing the action. A grape dispenser was placed in an adjacent corner. While the Passenger navigated toward the grape dispenser along a convoluted trajectory, its M1 and PMd neurons exhibited positional tuning that were like the tuning reported here, but they reflected the Passenger’s position with respect to the two other salient objects in the room: the grape dispenser and the Observer monkey. In control sessions where the location of the Observer monkey was swapped with that of the grape-dispenser, the position-tuning patterns of M1 and PMd also flipped accordingly. Although more experiments would need to be done to examine this effect in more detail, these results confirm that the place fields of M1 and PMd are defined by the arrangement of objects in the monkey’s environment.

Thus, our findings show that S1, M1, and PMd generate an allocentric representation of space, very much like hippocampal place cells. Additionally, our results are consistent with several studies conducted in different species showing allocentric spatial tuning of neurons outside the hippocampus. In the ferret primary auditory cortex, populations of neurons tuned to both allocentric and egocentric spatial location of sound were found^40^. The tuning properties were stable through several sessions. In the rat primary visual cortex^41^, subsets of neurons were found to be predictive of the upcoming visual stimulus in a position dependent manner. The position-dependency properties were found to be stable and the authors suggested that the visual cortex forms an internal representation of a visual scene based on spatial location, mediated by top-down projections from the hippocampus via the anterior cingulate cortex (ACC). In the mouse retrosplenial cortex (RSC)^42^, a subpopulation of neurons were found to express a sparse, orthogonal and continuous representation of a linear environment in a fashion highly similar to CA1 place-cells. The place-fields of these neurons were stable through time and could remap upon altering of spatial context. Place-cells in RSC were found to be most prevalent in the CA1 recipient layers, making the inheritance of spatial-information from the hippocampus a likely explanation. In the rat claustrum^43^, a subcortical structure that has extensive direct and indirect connections with the hippocampal-entorhinal structures, populations of neurons were found to exhibit activities similar to place-, boundary-, and object-cells in the hippocampal-entorhinal complex. The place-cells were found to have stable place-fields but did not remap according to distal cues or proximal objects. In rat lateral septum^44^, neurons were observed to represent context-specific location-selective firing. The spatial-selective activities were stable across days and remapped to different areas. The authors attributed their observations to direct projections from the hippocampus. All these reported areas have direct connections with the medial temporal hippocampal-enthorhinal complex.

Thus our results suggest that encoding of body position in space is much more distributed in the brain than previously thought. In this context, our findings expand considerably the extent of cortical circuits known to be involved in creating an animal’s spatial localization to include the premotor, and the primary motor and somatosensory areas, which are considered as hierarchically low processing cortical regions because they have no direct connectivity with hippocampal structures.

## Materials and Methods

All animal procedures were performed in accordance with the National Research Council’s Guide for the Care and Use of Laboratory Animals and were approved by the Duke University Institutional Animal Care and Use Committee.

### Study Design

The objective of this study was to examine the cortical representation during whole-body navigation. The experiments performed were described in detail in^9^, where we demonstrated whole-body navigation with control signals derived from neuronal ensemble recordings in multiple cortical area. Two adult rhesus macaques (monkey K and M) were used for the study. The two monkeys were chronically implanted with arrays of microwires in multiple cortical areas of both hemispheres (M1, S1, and PMd). Our multi-channel wireless recording system was employed to sample the spiking activities from hundreds of neurons in sensorimotor cortex simultaneously. Both monkeys learned to navigate in a room while seated in a mobile robotic wheelchair using their cortical activity as the navigation control signal. Cortical ensemble recordings were converted to steering commands for the robotic wheelchair based on linear decoding algorithms. Both monkeys successfully acquired the ability to steer the robotic wheelchair towards the reward dispenser using this BMI. They were able of achieving two-dimensional navigation with multiple starting positions and orientations of the wheelchair.

### Task Design

The monkeys were operantly conditioned to drive the wheelchair toward a food reward (grape dispenser). Each experimental session consisted of trials during which the monkeys navigated to the dispenser from one of three possible starting locations at the back of the room. Upon monkey arrival, the automated grape dispenser releasesd a grape (Fig. 1A). The wheelchair was then driven away from the grape dispenser to a new starting location and orientation. At the beginning of each experimental session we ran passive navigation trials, with the wheelchair executing preprogrammed routes identical from day to day. The passive trajectories were chosen empirically. Cortical neuronal responses during the passive navigation trials was used to obtain initial settings for two L2-norm Wiener Filters^45,46^, which decoded translational (forward and backward) and rotational (clockwise and counterclockwise) velocity from neuronal ensemble activity. Both translational and rotational velocities were expressed in chair-centered coordinates, not in room coordinates. Once the decoders were trained, we proceeded to brain-control trials during which the robotic wheelchair was steered directly by the cortical signals.

The translational velocity was limited to −0.28 to 0.28 m/s (negative values for backward movements and positive for forward), and rotational velocity was limited to −46 to 46 degrees/s (negative for counterclockwise, positive for clockwise). If the decoded velocity exceeded the limit, the command sent to the wheelchair would be set to the limit values.

The monkeys navigated in a 3.5-by-2.5 m experimental room, and the wheelchair’s dimension was 1.0-by-0.6 m. The actual drivable area was chosen to be 3.1-by-2.4 m, to ensure safety. When the robotic wheelchair was at the drivable area boundary, the program would stop the wheelchair if the decoded commands would have moved the robot across the boundary, and would only execute the decoded commands if the robot moved inside the drivable area. In the task, the three starting locations had coordinates of (0.86, 0.65), (1.0, 0), and (0.86, −0.65) meters, where (0,0) was the room center (Fig. 1A). During brain-control trials, when the wheelchair came close to the grape dispenser (docking range), the program would take over the control and park it automatically close to the dispenser to ensure the monkeys could comfortably obtain their rewards. When the monkeys came close to the grape dispenser, they could reach for the food. From video tracking and wheelchair localization, we compiled the range of distances from the dispenser where reach was initiated. The largest such observed distance in each session then defined the reach zone (Fig. 1A).

### Electrode Implantation

Monkey M was implanted with four multielectrode arrays for a total of 384 microelectrodes. Within each array, microelectrodes were grouped in two four-by-four, uniformly spaced grids each consisting of 16 microwire triplets of different lengths, for a total of 96 microelectrodes. Each hemisphere received two arrays, one each in the primary motor cortex (M1) and primary somatosensory cortex (S1). In the current experiment we used 256 channels in monkey M to record from neurons in the bilateral arm and leg areas of M1 and S1 (Fig. S1A).

Monkey K was implanted bilaterally with six multielectrode arrays containing 96 microwires each for a total of 576 microelectrodes. The arrays were implanted in the arm and leg representation areas of M1 and S1 and also in the bilateral premotor (PMd) cortices. In the current experiment we used 128 channels in monkey K to record from neurons in the S1, M1, and PMd in the right hemisphere (Fig. S1A).

### Experimental Apparatus

The experiment apparatus was composed of three components: (1) experiment control system (2), wireless recording system, and (3) robotic wheelchair. (Fig. S1B). The experiment control system controlled the experimental sequence, performed decoding of neuronal ensemble activity to wheelchair commands, dispensed grape, and handled the video tracking of both wheelchair and monkey head pose. The wireless recording system recorded neuronal ensemble activity from the monkey brain and sent the neuronal data to the experiment control system. The robotic wheelchair accommodated the monkey chair and received driving commands wirelessly from the experiment control system. The three components communicated with each other in a local network.

#### Experiment control system

The experimental control system controlled the flow of the experiment, which included controlling the experimental sequence such as starting a trial, initiating docking sequence, delivering grape reward, and ending a trial. The experimental control system also received the neuronal ensemble spiking activities from the wireless recording system, computed and sent navigation (velocity) commands to the robotic wheelchair, performed video tracking of the wheelchair and computed the wheelchair and monkey head pose. During brain-control trials, the system also ran BMI decoding algorithms that computed navigation commands for the robotic wheelchair from the neuronal ensemble activities.

The location and orientation of the wheelchair were tracked using an ASUS Xtion camera (640 × 480 pixels, 30 frames/sec) mounted on the ceiling of the experiment room. The video tracking software was written in C++ and built with OpenCV library. The software processed the video stream, and segmented the video frames to determine the robotic wheelchair location. The wheelchair orientation was determined based on the markers located at the front and back of the wheelchair. The wheelchair position signals were smoothed using a Kalman filter. Similarly, we put color markers on the cover of the monkey headcap to track its location and orientation the same way as we tracked the wheelchair. The position coordinate of the wheelchair was taken as the position of the center of the wheelchair.

#### Robotic wheelchair

The robotic wheelchair was a modified, commercially available mid-wheel drive model (Sunfire General) manufactured by Drive Medical. A Roboteq VDC2450 dual channel motor controller served as the interface between an onboard Raspberry Pi (RP) and the wheelchair motors. The RP received the computed motor commands through User Datagram Protocol (UDP) from the experiment control system, and the RP sent the commands to the Roboteq controller via serial data bus connection. As a safety feature, the Roboteq was programmed to stop the robotic wheelchair when the communication failed (i.e., the robotic wheelchair did not receive any motor commands for 1 s), or hit obstacles (i.e. the wheels failed to turn as current limit was set to 50 Amps). An emergency manual power disconnect was prominently placed on the vehicle that would disable the power to the wheels should a malfunction occur that requires a complete manual shut down. A secondary 2.4 GHz wireless control system also interfaced to the Roboteq controller and was used as a remote manual wireless control to assist in maneuvering the vehicle between experiments when it was not receiving commands from the experiment control system.

#### Wireless recording system

The wireless recording system was built -in-house and described in^47^. Briefly, the wireless recording system comprised the following: headstages, wireless-to-wired bridges, and client software. Each headstage includes digitizing amplifiers capable of recording from 128 microelectrode channels, microprocessors for digital signal processing and spike-sorting after receiving templates from the client software, and a 2.4 GHz wireless transceiver. The bridge received incoming radio packets from the headstage and transmitted them via UDP to the client software on the recording computer. The client software visualized the incoming data, performed spike sorting, and transmitted spike-timing data to the experimental control system.

The recording system was capable of detecting two neuronal units per recorded microelectrode channel. We recorded from 128 channels in monkey K and 256 channels in monkey M, yielding for each session 147.0 ± 10.2 neurons for monkey K and 157.1 ± 23.9 neurons for monkey M on average.

### Data Analysis

#### Data description

We ran 21 sessions with monkey K sessions and 28 sessions with monkey M, and sometimes two sessions in a day. Two sessions of monkey K and five sessions of monkey M were excluded from the analysis because (1) the sessions had less than 10 attempted BMI navigation trials (3 sessions) (2), technical issues occurred during recording (1 session) (3), a decoder was ill-trained and biased toward negative velocity (1 session), or (4) communication between the experiment control system and the Raspberry Pi on the robot failed frequently (two sessions). Ten sessions of monkey K were further excluded from analysis because (1) monkey head orientation was not recorded (eight sessions), and (2) total number of valid BMI trials were less than 10 (two sessions). A BMI trial was invalid if the localization algorithm lost track of the wheelchair, if the trial was paused, and if movement errors occurred. In the end, nine sessions from monkey K and 23 sessions from monkey M entered the analysis. On average, each analyzed session yielded 57.9+/−33.0 and 45.4+/−8.6 valid BMI navigation trials by monkey K and M, respectively.

Spike sorting was conducted on the first experimental session. However, neuronal units’ activity profile can vary between sessions due to microelectrode shifting and noise level changes. Minimal resorting was conducted in subsequent sessions. We excluded neurons that were not present in all analyzed sessions yielding 116 neurons in monkey K (27 in S1, 64 in M1, and 25 in PMd) and 124 neurons in monkey M (59 in S1 and 65 in M1).

#### Position-tuning diagrams

To visualize the position-tuning properties for each neural unit (Fig. 1B–G), we first aligned the measured wheelchair position to the neural activities. The wheelchair position measurement was down-sampled to 10 Hz, and the spike counts were binned into 100 ms time-bins. Next, each unit’s spike counts were z-scored by the 100 ms spike-counts of that unit, then smoothed by a 3-point moving average. The room was divided into 5cm-by-5cm spatial bins. Then, we sorted the normalized spike counts into this spatial grid based on the corresponding wheelchair position, and averaged the normalized spike counts that fell into the same bin. Lastly, missing values of the position-tuning diagram were filled in and Gaussian-smoothed (N = 7, *σ* = 3) by its neighboring spatial-bin values for illustration purposes. The position-tuning diagrams for different directions (Fig. 2A–D) were constructed similarly, where the alignment of neural activities and wheelchair position were conditioned on the monkey’s head orientation (*α*) or wheelchair orientation (β) being in a particular range.

#### Mutual information

Information theoretic analysis for single neurons have been widely used to quantify the amount of information conveyed by the firing rate of a neuron about any experimentally measured variable or combination of variables^10,24^. If each stimulus, *s*, were to evoke its own response, *r*, then on measuring *r* one would confirm *s*, and thus gain $I(s)=-lo{g}_{2}{\rm{P}}(s)$ bits of information, where *P*(*s*) is the a priori probability of occurrence of a particular stimulus *s*. If instead, the same response can be invoked by several stimuli with different probabilities, this probabilistic stimulus-response relation can be expressed by a joint probability distribution *P*(*s*,*r*). The information about *s* gained by knowing *r* can be evaluated by

1 $$I(s,R)={\sum }_{r}P(r|s)lo{g}_{2}\frac{P(s|r)}{P(s)}$$

Averaging over different stimuli *s* in the set of stimuli *S*, the average information gain (MI) about the set of stimuli *S* present in the neuronal spike data *R*, (where *R* denotes the set of responses *r*) is

2 $$I(S,R)={\sum }_{s}P(s)I(s,R)={\sum }_{s,r}P(s,r)lo{g}_{2}\frac{P(s,r)}{P(s)P(r)}$$

To obtain the amount of information a neuron’s spike train conveys about the wheelchair’s spatial location pre-docking, we first divided the experimental room into 0.4m-by-0.4 m grids (set of stimulus *S*). Wheelchair position measurement and spike counts were aligned and binned into 100 ms time-bins. For each neuronal unit, the set of responses *R* is the distinct spike-count observed in a 100 ms time-bin. Then, we sorted the spike-counts into the spatial-grid based on the wheelchair position and constructed joint-probability tables between all pairs of stimulus and response for each neuron. The amount of information each unit’s neuronal activity conveys about spatial location in bits/100 ms is then computed according to equation (2). We further bias-corrected these MI values following the procedures in^48^ by subtracting a correction-term

3 $${C}_{1}=\frac{1}{2Nln2}({N}_{s}-1)({N}_{R}-1)$$

from each value, where *N*_*S*_ is the number of distinct stimulus, and ${N}_{R}$ is the number of distinct neuronal response. We set a neuron’s final mutual-information value to 0 if its bias-corrected mutual-information value was less than 0, or if its ${C}_{1}\,$value was greater than 1. MI for orientation parameters ($\alpha$ and $\beta$) were computed similarly, with the set of stimulus *S* as $\alpha$ and $\beta$ values discretized into eight angular bins of 45 degrees each.

We chose the position and angular stimulus discretization to ensure adequate sampling of the stimulus space – the number of discrete bins must not be too high for limited sampling effects, even after the correction procedure, to bias information estimated based on limited numbers of trials^49^.

To test the significance of the mutual-information values, we performed permutation tests. We shifted the alignment of the wheelchair position, $\alpha$ or $\beta$ a random interval between 10 to 50 seconds with respect to the neuronal activities and computed the resulting bias-corrected MI values. This was done 1000 times to obtain distributions of permuted MI values. From this permuted distribution the p-value of the actual MI value can be obtained for each neuron. The p-values were then corrected by false-discovery rate (FDR) and considered significant if the corrected value is under 0.05.

#### Generalized Additive Model (GAM)

A generalized additive model is an extension generalized linear model (GLM) that involves a weighted sum of smooth functions of covariates^26^. In GAM, the relationships between the individual predictors and the dependent variable combine generalized linear models with the benefits of non-parametric smoothing. Nonparametric here means that the shape of predictor functions were fully determined by the data as opposed to parametric functions that were defined by a typically small set of parameters. This provides the advantage of having the ability to capture nonlinear relationships between the response variable and the covariates (otherwise known as “tuning function”), while still having the interpretability of a linear model. Mathematically, a GAM has the form of

4 $$g(E(y))={\sum }_{n=1}^{N}{f}_{n}({x}_{n})+{\sum }_{m=1}^{M}{x}_{m}+{\epsilon }$$

where y is the response variable, $E(y)\,$denotes the expected value, $g(.)\,$denotes the link function that links the expected value to the predictors ${x}_{n}\,$and ${x}_{m}$. The first summation contains the nonparametric terms where ${f}_{n}\,$are smooth non-linear functions describing the relationship between ${x}_{n}\,$and the response variable. The second summation contains the linear terms and ${\epsilon }\,$represents the noise term – these are identical to those in a GLM.

In GAM, each ${f}_{n}$ is represented by a linear combination of spline basis functions that describes the predictor-response relationship as a piecewise polynomial. Penalized regression splines are used to prevent overfitting of the spline functions ${f}_{n}$. All GAMs in this work were fitted using the *mgcv* R-package^27^. To quantify the dependence of firing rate of individual neurons on the different parameters, we implemented the following full model with *mgcv*:

5 $$\begin{array}{lll}g(E(Y)) &  \sim  & ti(x,y)+ti(v,\omega )+ti(\alpha )+ti(\beta )\\  &  & +ti(x,y,v,\omega )+ti(x,y,\alpha )+ti(x,y,\beta )\\  &  & +ti(v,\omega ,\alpha )+ti(v,\omega ,\beta )\\  &  & +ti(\alpha ,\beta )\\  &  & +S(Y(t-1))+S(Y(t+1))\end{array}$$

The response variable Y is the number of spikes in 100 ms time bins, Y(t − 1) and Y(t + 1) represent the number of spikes shifted in time by one time bin. The link function $g(\,\cdot \,)\,$was chosen to be log and the response was fitted assuming quasipoisson conditional distribution. The predictors $x$, $y$, $v$, $\omega$, $\alpha$, and $\beta$ represent the wheelchair’s x-position, y-position, linear velocity, rotational velocity, monkey head angle and wheelchair angle, respectively. The functions $S()\,\,$and $ti()$ indicate nonparametric smoothing is to be used. $ti()$ was used because both the main-effects and the interaction between the main-effects were included in the model. In equation(5), the terms on the right-hand stand of the first line represents the main-effects of position, wheelchair velocity, head, and wheelchair angles, respectively – these can be seen as the “tuning function” or “tuning curve” of the neuron to these predictors. Note that the effects of both position and velocity main-effects are to be modeled with 2D smoothing functions, as indicated by the bivariate functional form. The second line of equation (5) represents all the interaction terms between position and the other three predictors. Since the main-effect of position is assumed to be a bivariate function, these terms represent the interaction between a bivariate function with either a bivariate (e.g. $ti(x,y,v,\omega )$) or univariate (e.g. $ti(x,y,\alpha )$) function. The third and fourth line of equation (5) include all the interaction terms between velocity and orientation parameters, and between the orientation parameters, respectively. The last line of equation (5) includes the effects of previous and next bins of spike-counts – these terms are included for a more complete model specification because spike-counts are known to and indeed exhibited autocorrelation^50^. This full model specification was deemed appropriate after examining diagnostic plots.

To assess the relative importance of a group of covariates, we fitted partial models from the full model by removing those covariates. For example, to assess the effects of position on a neuron’s activities, we constructed the partial model by removing the $ti(x,y)$ term from equation (5). F-test was conducted to check if the full-model is significantly better than the partial-model, i.e. if a neuron is “tuned” to position. To quantify the effects of position, or tuning-depth (TD), we calculated the McFadden’s pseudo R-squared^51^ as

6 $$1-\frac{{D}_{F}}{{D}_{P}}$$

where ${D}_{F}$ and ${D}_{p}\,$are the deviance of the full and partial-models, respectively. Deviance is analogous to the sum of squares of residuals in regression by ordinary least squares (OLS) and measures the lack-of-fit of a model. The pseudo R-squared quantity is analogous to partial R-squared in OLS and measures how much of the neuronal activities can be explained by position. The full and partial model differ only in the main-effect term of position, and the interaction terms between position and all other predictors are kept so that equation (6) measures only the contributions to the neuronal activities from position alone, excluding those due to predictor correlations. TD to position, head-direction $\,\alpha$ and wheelchair direction *β* were calculated this way for all neurons.

#### Place-field heatmap

The GAM fitting process produces the neurons’ position tuning-functions as the bivariate position main-effect terms. For each neuron, we defined its place field as the room locations where the neuron’s tuning function exceeded the median value over all room locations. A histogram of place-fields was constructed from all position-preferring cells’ place-fields and normalized by the number of position-preferring cells in each session. These session histograms were then averaged to produce the final heatmap.

#### Preferred-direction histogram

The GAM fitting process produces the neurons’ α- and $\beta$- tuning curves as the univariate cyclical $\alpha \,$and $\beta \,$ main-effect terms. For each neuron, we defined its preferred directions as the argmax of the tuning curves. Histograms of the preferred directions were constructed from all orientation-preferring cells’ tuning curves and normalized by the number of orientation-preferring cells in each session. These session histograms were then averaged to produce the final histogram.

#### Consistency of tuning properties

To analyze the consistency of individual neurons’ tuning properties within a session and across multiple sessions, we first excluded units that were resorted in any of the analyzed sessions. Twelve cells were excluded in monkey K and 15 cells were excluded in monkey M. We then selected from each monkey neurons that were highly tuned to either spatial location or orientation in at least 70% of analyzed sessions. For each neuron, we constructed a three-dimensional histogram where each bin represents a unique combination of (x-position, y-position, head-direction $\,\alpha$). The neuronal activities falling within each histogram bin were then averaged. This trivariate tuning function allowed us to compare position- and orientation-tuning properties at the same time. To ensure adequate sampling, we used 0.1 m discretization for x- and y-positions, and divided head-direction into four bins of 90 degrees each.

To evaluate how similar the tuning properties were between two sessions, we obtained the pair-wise consistency index by first calculating the Pearson correlation between each neuron’s trivariate tuning function in the corresponding sessions and then averaged that from all neurons. The within-session consistency index was calculated similarly by dividing each session into two equal continuous blocks and obtaining a trivariate tuning function for each neuron in each block.

To calculate the significance of the consistency index, we performed permutation tests by shuffling the sessions’ neuronal data by a random time between 10 and 50 seconds with respect to the covariates and computing all pairwise- and within-session consistency indices. This procedure was repeated 1000 times to obtain distributions of permuted consistency indices. From this permuted distribution the p-value of the actual consistency index value was obtained. To compare pairwise- and within-session consistency of tuning properties, we performed the Wilcoxon rank-sum test between pairwise- and within-session consistency indices. The consistency indices were considered to be statistically different if the test rejected the null hypothesis at alpha level of 0.05.

#### Off-line position and orientation decoders

For monkey K, nonlinear autoregressive neural network with external input (NARX) decoders were trained and tested off-line to predict position, head-direction *α* and wheelchair direction *β*. NARX differ from the common feed-forward neural network in that the decoded outputs are fedback to the input layer^52^. This architecture is suitable for time-series prediction because it utilizes the information present in the data’s autocorrelation structure. Position decoders had a single hidden layer with 30 artificial neurons (nodes) fully connected to two output nodes corresponding to the x- and y-position. $\alpha$ and *β* decoders each had a single hidden layer with 10 nodes fully connected to two output nodes corresponding to the sine and cosine of the target angle. Decoder output at time $t$ was computed from five 100ms-bins of z-scored spike-counts (each bin correspond to time $t\,$to $t-4$) of the chosen neurons, and three past predictions (time $t-1\,$to $t-3$). For monkey M, feed-forward neural network (FFN) decoders were used where the decoded outputs were not fedback to the input, and all other network configurations stayed the same. We found this produced less noisy predictions for monkey M. The NARX and FFN decoders were implemented using MATLAB 2014b’s (Mathworks, Boston, Massachusetts) neural network toolbox. All hidden layer nodes had hyperbolic-tangent transfer function, the output layer nodes had linear transfer function. All decoders were trained with conjugate gradient descent with Fletcher-Reeves updates^52^ with early-stopping after 15 validation failures.

For each session we performed 10-fold cross-validation on the brain-controlled trial data pre-docking with five retrainings per fold, yielding for each fold a best performing decoder for each parameter. The performance was measured by either Pearson’s correlation coefficient (CC) between the predicted and actual values for orientation parameters, or mean prediction error (MPE) for position. MPE is defined as the average distance between the predicted and actual locations. The median CC (Fig. S4) or MPE (Fig. 5A) across all folds were taken as a session’s decoding performance.

To assess the chance decoding performance, the decoders were trained and tested on shuffled session data constructed by first shifting the neuronal data by a random amount with respect to the covariates, and then shuffling the resulting time series (to destroy the autocorrelation structure). For each session-population-type combination, this was done 100 times to obtain 95% confidence intervals for chance decoding performance, from which we obtained the p-value of actual decoding performance.

To visualize the location prediction performance over space (Fig. 5B–G), we first concatenated the prediction error, defined as the distance between the predicted and actual locations, from all 10 folds of testing data. These prediction errors were then sorted into the spatial-grid defined in *position-tuning diagram* and averaged within each bin. Lastly, spatial bins with missing values were filled in and Gaussian-smoothed (N = 5, $\sigma$ = 2) by the values of its spatial-bin neighbors.

#### Reach activity

Population peri-event time histograms (PETHs) aligned on the wheelchair entering the reach zone was constructed from smoothed z-scored spike-counts (100 ms time bins, 3-point moving average) from all brain-controlled trials in a session. Neuronal units were sorted by the slope of its peri-event activities, as calculated by ordinary linear regressions (Fig. S5A–B).

#### Reach decoder

For brain-controlled trials in each session, we labeled all neuronal activities inside the reach-zone as reach-related, and all neuronal activities outside as navigation-related. There was roughly nine times as many navigation-related time points as reach-related in each session, making the data-set highly imbalanced. Principle component analysis (PCA) was first conducted on the smoothed z-scored spike-counts (100 ms time-bins, 3-point moving average). Ensemble decision tree classifiers were then used to classify reach activities from the top five PCs. Each ensemble classifier had 400 decision trees^53^ with minimum leaf-size of 20, and was trained using MATLAB 2014b’s *fitensemble* function. Random under sampling (RUSBoost)^54^ was chosen to fit the ensemble classifiers. For each session, we performed 5-fold cross-validation, yielding for each fold an ensemble classifier. The performance of each classifier is measured by Cohen’s $\kappa$^28,49^ between the actual and predicted labels (reach or not) on the testing data. Cohen’s $\kappa$ takes into account of the distribution of the actual labels and is an appropriate measure for imbalanced data classification. The $\kappa$ values for all five folds were averaged to obtain each session’s reach-classification performance.

To visualize reach-classification in space (Fig. S5C–H), we first concatenated the predicted probabilities (scores) of reach from all five folds of testing data in a session. The score of reach at each time point is the mean terminal leaf probability across all 400 decision-trees in the ensemble classifier at that time. We then sorted the scores into the spatial-grid as defined in *position-tuning diagram* and averaged the scores that fell into the same bin. Lastly, spatial bins with missing values were filled in and Gaussian-smoothed (N = 5, $\sigma$ = 2) by the mean scores of its spatial-bin neighbors.

### Statistical analysis

To assess the difference between preferred TDs among brain regions within the same monkey, we used the Kruskal-Wallis test with Tukey’s multiple comparison on neurons pooled over all sessions. To assess the difference in proportion of neurons tuned to space or orientation among brain regions within the same monkey, we used Friedman’s test with Tukey’s multiple comparison, where sessions were a repeated measure. To assess the difference between preferred TDs between different monkeys, we used Wilcoxon rank-sum test on neurons pooled over all sessions for each monkey. To compare the position and orientation decoding performance from using different neuronal populations, we used Kruskal-Wallis test with Tukey’s multiple comparison. To assess the uniformity of the population place-field-heatmap (Fig. 3A,B) and preferred-direction histograms (Fig. 3C–F), we used the Kolmogorov-Smirnov test to compare those distributions against the uniform distribution. To assess the decoding of reach-state taking into account o the ensemble size for reach state decoding, we used nonparametric ANCOVA. We consider a statistical test to be significant if p < 0.05, and all Tukey’s multiple comparison had α level of 0.05.

## Electronic supplementary material

Supplementary Figures and Tables
