# Supplementary material for: Place Cell-Like Activity in the Primary Sensorimotor and Premotor Cortex During Monkey Whole-Body Navigation
Source: Sci Rep. 2018 Jun 15;8:9184. doi: 10.1038/s41598-018-27472-4 (PMC6003955; doi:10.1038/s41598-018-27472-4)
Supplement: Supplementary file 1 — Supplementary Figures and Tables [file 41598_2018_27472_MOESM1_ESM.pdf]

Supplementary Information

PLACE CELL-LIKE ACTIVITY IN THE PRIMARY SENSORIMOTOR AND PREMOTOR CORTEX DURING MONKEY WHOLE-BODY NAVIGATION

A. Yin, P.H. Tseng, S. Rajangam, M.A. Lebedev, M.A.L. Nicolelis

Supplementary Files:

Figures S1 to S5
Tables S1 to S3

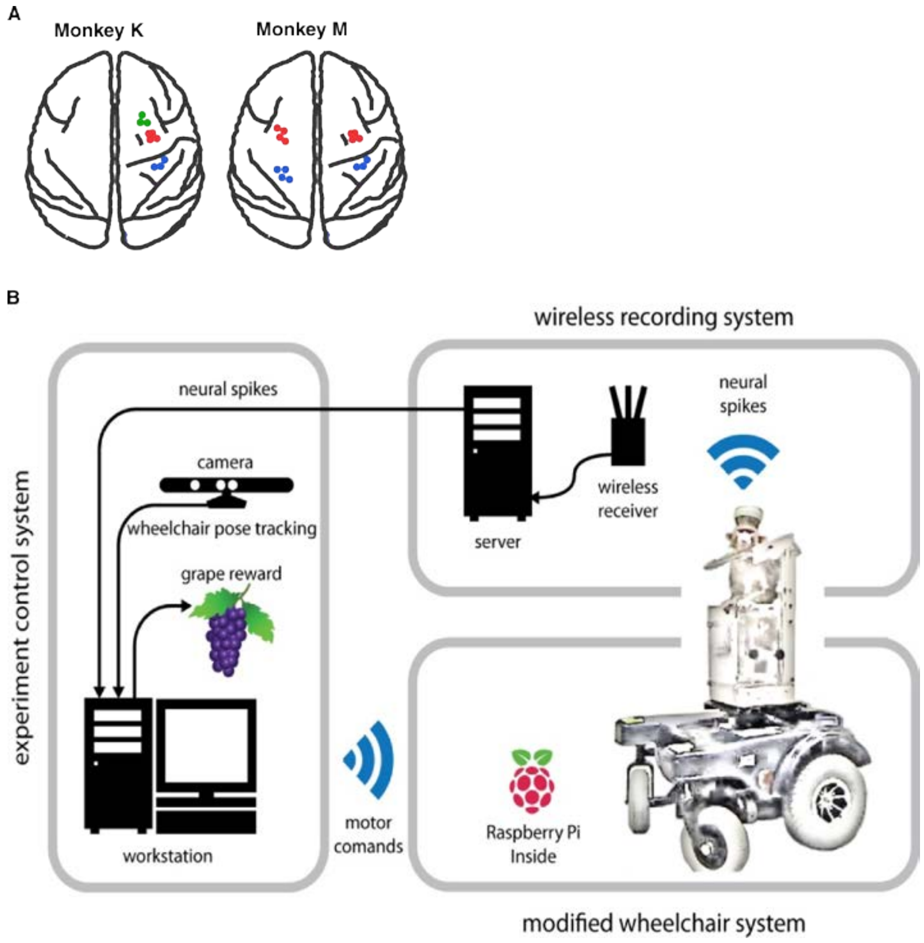

Figure S1. Recorded cortical sites and schematics of experimental design (A) Schematic of the brain regions from which we recorded neuronal units. Blue dots correspond to units in S1, red dots to units in M1 and green to units in PMd. **(B)** The driving experiment was composed of three systems. The experiment control system controls the experiment flow, decodes monkey's neural signals, tracks the pose of the wheelchair and delivers the grape reward. The wireless recording system receives the monkey's spiking activities from the monkey's head stage, and sends the activities to the experiment control system. The modified wheelchair system executes the (decoded) wheelchair movement commands from the experiment control system.

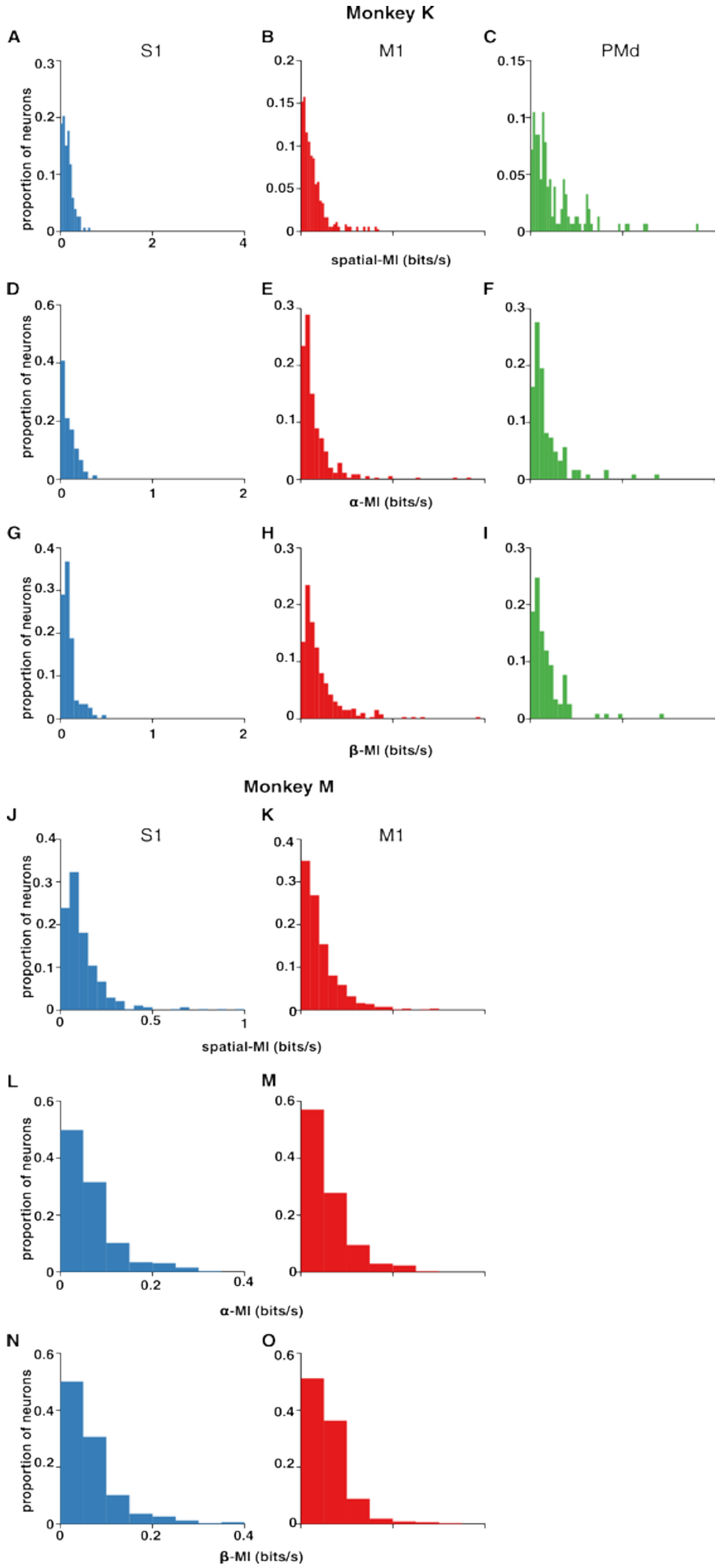

Figure S2. Distributions of bias-corrected mutual-information are plotted for neurons in all sessions, corresponding to spatial-location (A-C, J-K), α(D-F, L-M), and β(G-I, N-O); for monkey K (A-I) and monkey M (J-O); and categorized by brain regions – S1 (A, D, G, J, L, N), M1 (B, E, H, K, M, O), and PMd (C, F, I). Only neurons with significant bias-corrected mutual-information values are plotted.

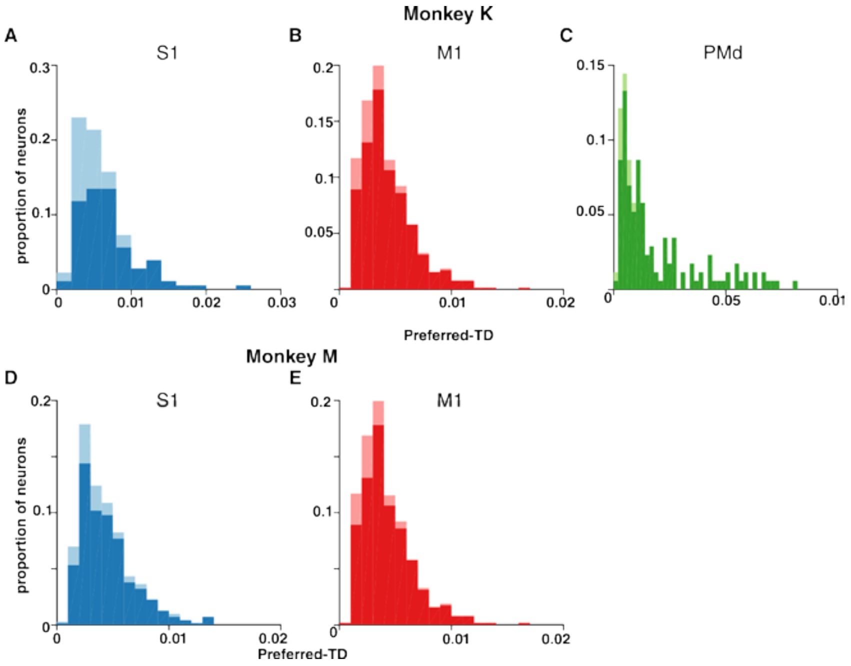

Figure S3. Distribution of preferred tuning-depth for neurons in all sessions significantly tuned to either spatial-location or orientation, for monkey K (A-C) and monkey M (D-E), categorized by brain regions – S1 (A, D), M1 (B, E), and PMd (C). Bars with darker shades represent the preferred tuning-depth values for neurons that prefer spatial-location, and bars with lighter shades represent the preferred tuning-depth values for neurons that prefer orientation.

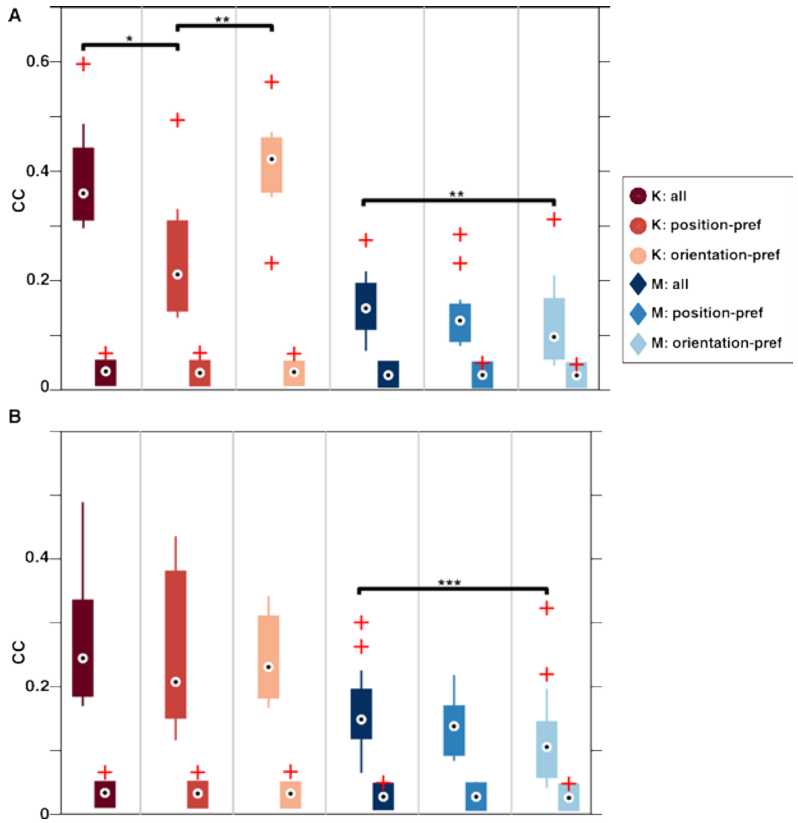

Figure S4. Decoding of orientation from neuronal ensemble activity. (A) Box plots showing the distribution of decoding accuracy and chance decoding accuracy (left and right boxes within each group, respectively) measured by Pearson's product moment correlation coefficient (cc) between the actual and predicted α values in monkey K and monkey M using all neurons, spatial-preferring and orientation-preferring neurons. Only sessions with decoding accuracy significantly above chance are shown. The distributions of chance-level decoding accuracy are constructed from the lower-end of the 95% confidence interval of the selected sessions' chance-level error. (*) indicates significant p-value < 0.05; (**)=p<0.01; (***)=p<0.001; for post-hoc Tukey's multiple comparison following Kruskal-Wallis test. **(B)** Decoding accuracy for β, conventions as in (A).

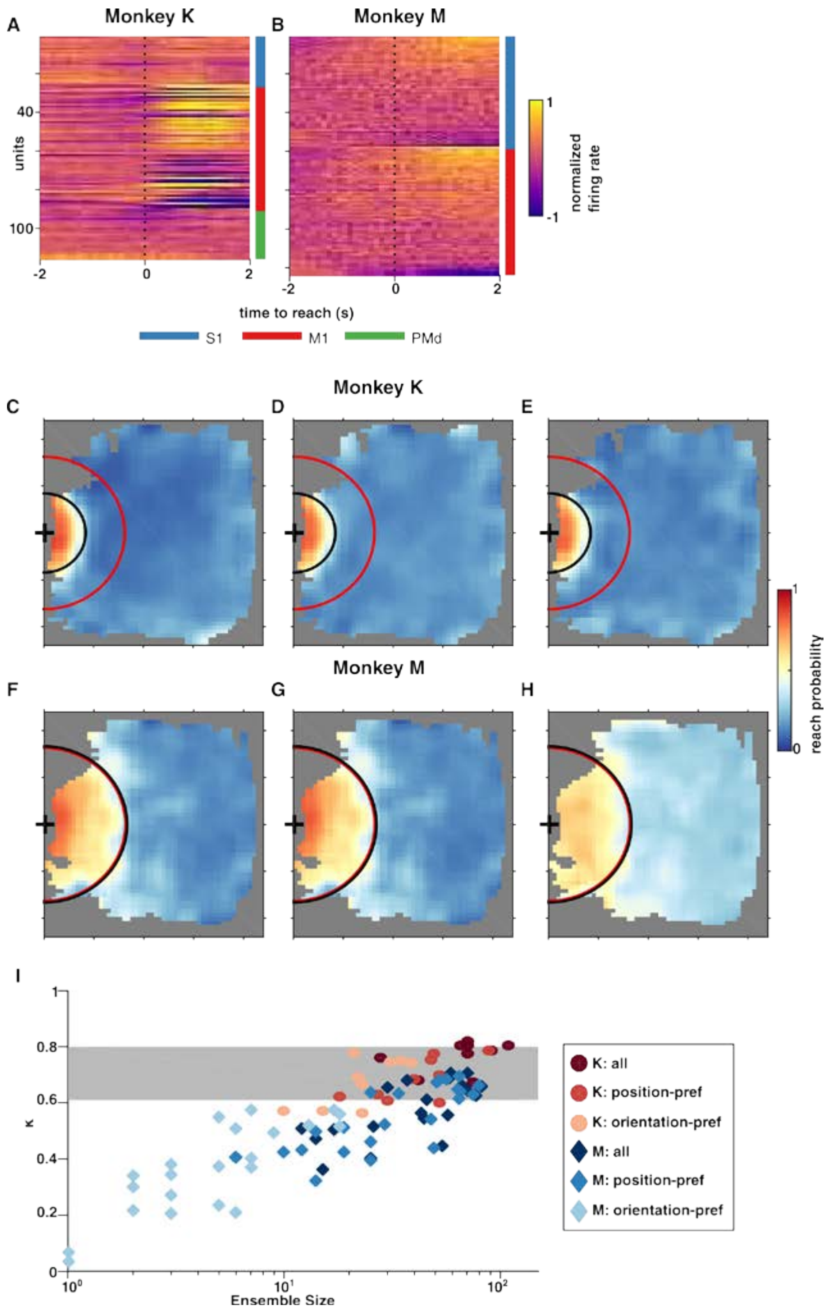

Figure S5. Reach-related activity and its classification. (A-B) Color-coded population perievent time histograms (PETHs) for monkey K (A) and monkey M (B) neuronal populations (all neurons entered into GAM analysis). Each horizontal line represents trial-average z-scored firing rate for a neuronal unit. The PETHs are centered on arm-reaching onset. The neurons are grouped by cortical areas. For each area, individual-neuron PETHs are sorted by the peri-movement discharge rate. **(C-H)** Color plots of classification of arm-reaching state as a function of room location for monkey K (C-E) and monkey M (F-G), based on activities from all neurons (C, F), subpopulation of highly-tuned spatial-preferring (D, G), and subpopulation of highly-tuned orientation-preferring (E, G) cells, for one representative session for each monkey. Color represents probability of classification. **(I)** Bar plots showing accuracy of reach-state decoding for monkey K (left) and monkey M (right), using all tuned neurons, and subpopulation of highly-tuned spatial-preferring and orientation-preferring neurons. The accuracy is measured by Cohen's kappa between the predicted and the actual reach classifications. Each bar shows the median accuracy

over all sessions and the error bars indicate the interquartile range. (**) indicates significance at $p < 0.01$ for post-hoc Tukey's multiple comparison following Kruskal-Wallis test.

Table S1: Percentages of neurons with significant MI to parameters by cortical area

	Spatial location	α	β	Spatial or orientation	Spatial and orientation
Monkey K					
S1 (n=27)	63.0±20.6%	31.3±22.1%	48.1±22.8%	73.3±21.1%	44.0±21.7%
M1 (n=64)	62.8±20.3%	60.1±20.1%	69.6±16.2%	84.7±11.6%	55.2±20.9%
PMd (n=25)	68.0±15.7%	54.7±15.4%	52.0±16.2%	76.9±12.3%	56.9±14.4%
All neurons (n=116)	64.0±18.8%	52.2±18.6%	60.8±17.1%	80.4±12.9%	53.0±19.3%
Monkey M					
S1 (n=59)	35.4±14.7%	23.8±18.3%	22.4±18.6%	47.2±18.4%	19.8±15.4%
M1 (n=65)	37.4±17.7%	23.1±19.5%	21.2±20.6%	47.9±19.5%	20.1±18.1%
All neurons (n=124)	36.5±16.1%	23.4±18.7%	21.8±19.4%	47.5±18.7%	20.0±16.6%

Numbers represent session mean+/-std percentages.

Table S2: Percentage (mean±std) of neurons with significant tuning to different parameters by brain regions for Monkey K

	S1 (n=19.8±5.7)	M1 (n=54.27±.4)	PMd (n=19.2±3.1)	All regions (n=93.2±15.0)
Spatial	64.5±18.3%	39.2 23.9%	84.7±8.2%	53.7±18.8%
Orientation	48.0±23.5%	49.7±17.2%	48.2±22.9%	49.0±15.9%
Spatial and Orientation	36.1±20.1%	24.0±16.9%	43.1±24.8%	30.4±17.6%
Spatial or Orientation	76.3±20.8%	64.9±22.1%	89.8±5.1%	72.3±16.3%
Spatial-preferring	53.1±16.7%	32.1±18.9%	81.9±6.5%	46.7±15.5%
Orientation-preferring	23.2±15.6%	32.8±11.6%	7.8±4.3%	25.67±.6%

Only neurons with significant MI to orientation or space entered the tuning analysis. Numbers in parentheses indicate the session mean±std numbers of neurons that entered the analysis.

Table S3: Percentage (mean±std) of neurons with significant tuning to different parameters by brain regions for Monkey M

	S1 (n=27.8±10.8)	M1 (n=31.3±12.7)	All regions (n=59.0±23.1)
Spatial	68.1±21.9%	66.2±22.7%	67.0±21.1%
Orientation	33.4±18.7%	32.1±21.8%	32.6±19.5%
Spatial and orientation	26.5±18.0%	25.2±19.8%	25.7±18.5%
Spatial or orientation	75.0±21.2%	73.1±22.2%	73.9±20.2%
Spatial-preferring	63.5±22.2%	62.6±22.8%	62.9±20.8%
Orientation-preferring	11.5±10.3%	10.4±9.6%	10.9±9.0%

Only neurons with significant MI to orientation or space entered the tuning analysis. Numbers in parentheses indicate the session mean±std numbers of neurons that entered the analysis.
